# Utilization capability of sucrose, raffinose and inulin and its less-sensitiveness to glucose repression in thermotolerant yeast *Kluyveromyces marxianus *DMKU 3-1042

**DOI:** 10.1186/2191-0855-1-20

**Published:** 2011-07-19

**Authors:** Noppon Lertwattanasakul, Nadchanok Rodrussamee, Suprayogi, Savitree Limtong, Pornthap Thanonkeo, Tomoyuki Kosaka, Mamoru Yamada

**Affiliations:** 1 Applied Molecular Bioscience, Graduate School of Medicine, Yamaguchi University, Ube 755-8505, Japan; 2 Department of Microbiology, Faculty of Science, Kasetsart University, Bangkok 10900, Thailand; 3 Department of Biotechnology, Faculty of Technology, Khon Kaen University, Khon Kaen 40002, Thailand; 4 Department of Biological Chemistry, Faculty of Agriculture, Yamaguchi University, Yamaguchi 753-8515, Japan

**Keywords:** *Kluyveromyces marxianus*, inulinase, glucose repression, *INU1*, *MIG1*

## Abstract

*Kluyveromyces marxianus *possesses a useful potential to assimilate a wide variety of substrates at a high temperature, but the negative effect by coexisting glucose is critical for utilization of biomass containing various sugars. Such a negative effect on the activity of inulinase, which is the sole enzyme to hydrolyze sucrose, raffinose and inulin, has been demonstrated in *K. marxianus *without analysis at the gene level. To clarify the utilization capability of sucrose, raffinose and inulin and the glucose effect on inulinase in *K. marxianus *DMKU 3-1042, its growth and metabolite profiles on these sugars were examined with or without glucose under a static condition, in which glucose repression evidently occurs. Consumption of sucrose was not influenced by glucose or 2-deoxyglucose. On the other hand, raffinose and inulin consumption was hampered by glucose at 30°C but hardly hampered at 45°C. Unlike *Saccharomyces cerevisiae*, increase in glucose concentration had no effect on sucrose utilization. These sugar-specific glucose effects were consistent with the level of inulinase activity but not with that of the *KmINU1 *transcript, which was repressed in the presence of glucose via KmMig1p. This inconsistency may be due to sufficient activity of inulinase even when glucose is present. Our results encourage us to apply *K. marxianus *DMKU 3-1042 to high-temperature ethanol fermentation with biomass containing these sugars with glucose.

## Introduction

Glucose-mediated negative control in the budding yeast *Saccharomyces cerevisiae *is a model system for transcriptional repression (Ronne 1995; Entian and Schuller 1997; Gancedo 1998). This control, called glucose repression, physiologically occurs when glucose coexists as one of carbon sources, by which cells shut down the transcription of a specific set of genes for respiration, gluconeogenesis and the metabolism of alternative carbon sources, which may allow cells to perform rational energy consumption.

*ScSUC2 *in *S. cerevsiae *is exclusively and strongly regulated by glucose. Results of extensive genetic analyses with mutants defective in glucose repression and derepression and with extragenic suppressors as well as results of protein-protein interaction studies have led to an understanding of the regulation mechanism of *ScSUC2 *(Johnston and Carlson 1992; Entian and Schuller 1997), in which two glucose specific effectors, ScMig1p and ScMig2p, are vitally involved (Nehlin and Ronne 1990; Luftiyya and Johnston 1996). *KlINV1 *for invertase in *Kluyveromyces lactis *is also under the control of glucose repression, but in contrast to that of *ScSUC2*, its repression is independent of KlMig1p (Georis et al. 1999).

Invertase secreted from *S. cerevisiae *cells resides mainly in the cell wall to perform its physiological function, cleavage of sucrose molecules diffusible into the cell wall (Nam et al. 1993). Such specific localization of invertase may be ecologically beneficial for efficient scavenging of hydrolyzed products. Similarly, the cell-wall retention of inulinase may be advantageous for sucrose utilization in *K. marxianus*. However, this may not be the case for raffinose or inulin utilization because both sugar molecules hardly penetrate into the cell wall (Phelps 1965; Scherrer et al. 1974) and must therefore be hydrolyzed outside the cell wall.

Production of inulinase has been extensively investigated in *K. marxianus *(Cruz-Guerrero et al. 1995; Kalil et al. 2001; Singh et al. 2007). The investigation was mainly focused on optimization of its production under various conditions including operating parameters such as pH, temperature, agitation and aeration in addition to the culture medium, but the results were not sufficient to provide a clear picture of its regulation mechanism. As a consequence, conflicting opinions regarding expression of the enzyme have accumulated. It was demonstrated that inulinase synthesis is under the control of induction by its substrate with catabolic repression in *K. fragilis *and *K. bulgaricus *(Grootwassink and Fleming 1980; Grootwassink and Hewitt 1983), of induction without catabolic repression in *K. marxianus *UCD (FST) 55-82 (Parekh and Margaritis 1985) or of induction with catabolic repression in *K. marxianus *CBS 6556 (Rouwenhorst et al. 1988). On the other hand, other strains in the same species exhibit no induction by a substrate (Cruz-Guerrero et al. 1995; Schwan et al. 1997). Furthermore, Gupta et al. (1994) reported that glucose is responsible for catabolic repression, whereas sucrose and fructose act as weaker inducers than inulin in *K. fragilis*. However, all of these reports focused on the enzymatic activity of inulinase in the culture medium or cell wall fraction but not on expression at the transcriptional level. *KmMIG1 *has been cloned and characterized in *K. marxianus *SGE11 (Cassart et al. 1997), revealing that its physiological role is similar to that of *ScMIG1 *in *S. cerevisiae*; that is, KmMig1p represses the expression of *KmINU1 *as a counterpart of *ScSUC2 *in *S. cerevisiae *and was shown to be fully functional when expressed in *S. cerevisiae*.

Aiming at the realization of high-temperature fermentation as a beneficial and economical technology, utilization capability of various sugars derived from hemicellulose and ethanol productivity have been shown in thermotolerant *K. marxianus *DMKU 3-1042 at a relatively high temperature (Rodrussamee et al. 2011). The effect of glucose repression on sugar utilization in the organism, which becomes a critical point for application of biomass containing various sugars, has been shown to be more evident under a static condition. In this study, to determine the regulation mechanism of inulinase via glucose in *K. marxianus *DMKU 3-1042, we compared the fermentation capabilities of its substrates, sucrose, raffinose and inulin, in the presence and absence of glucose at different temperatures under a static condition, and we examined the effects of glucose on the transcripts of *KmINU1 *and *KmMIG1 *and on the production and secretion of inulinase. Detailed analyses reveal that *K. marxianus *DMKU 3-1042 is useful for high temperature fermentation with biomass constituted of these sugars and glucose.

## Materials and methods

### Materials

Oligonucleotide primers were synthesized by Proligo Japan (Tokyo). Other chemicals were all of analytical grade.

### Strains, media and culture conditions

Yeast strains used in this work were *K. marxianus *DMKU 3-1042 strain, which has been deposited in the NITE Biological Resource Center (NBRC) under the deposit number NITE BP-283 (Limtong et al. 2007), and *S. cerevisiae *BY4743 (*MAT*a/α *his3*Δ*1/his3*Δ*1 leu2*Δ*0/leu2*Δ*0 LYS2/lys2*Δ*0 met15*Δ*0/MET15 ura3*Δ*0/ura3*Δ*0*). Media used were YP (1% w/v yeast extract and 2% w/v peptone) supplemented with different carbon sources: YPD, with 2% w/v glucose; YPSuc, with 2% w/v sucrose; YPRaf, with 2% w/v raffinose; YPInu, with 2% w/v inulin; YPGal, with 2% w/v galactose; YPFrt, with 2% fructose; YPDSuc, with 2% w/v glucose and 2% w/v sucrose; YPDRaf, with 2% w/v glucose and 2% w/v raffinose; YPDInu, with 2% w/v glucose and 2% w/v inulin; and YPDGal, with 2% w/v glucose and 2% w/v galactose. If required, 0.01% w/v 2-deoxyglucose (2-DOG) was added to the medium. Cells grown in YPD medium at 30°C for 18 h were inoculated into a 100-ml batch culture medium in a 300-ml Erlenmeyer flask and incubated under a static condition at 30°C or 45°C. The culture flasks were shaken to make cell density homogeneous before samples were taken for measurement as times indicated.

### Analytical methods

Cell growth was determined by means of periodical optical density (660 nm) measurement. Concentrations of glucose, ethanol, sucrose, raffinose, inulin, fructose, melibiose and galactose during fermentation were determined at 35°C by an HPLC system consisting of an L-2130 Pump, L-2490 Refractive Index Detector, L-2200 Autosampler, L-2350 Column oven, and Hitachi Model D-2000 Elite HPLC System Manager, equipped with a GL-C610-S Gelpack^® ^column (Hitachi Chemical, Tokyo, Japan) using distilled water from an RFD240NA Water Distillation Apparatus (Aquarius, ADVENTEC^®^, Japan) as a mobile phase at a flow rate of 0.3 ml/min.

To examine production and distribution of inulinase, inulinase activity was measured at 50°C as described previously (Rouwenhorst et al. 1988) except that the initial rate of reducing sugar released was determined by the colorimetric 3,5-dinitrosalicylic acid method (Miller 1959). Cells were grown at 30°C or 45°C as described above and the culture at 6 h was subjected to a low-speed centrifugation to separate supernatant and precipitate fractions. The latter was suspended in 0.1 M acetate buffer (pH 4.5). Both fractions, called supernatant and cell fractions, were then used for inulinase assay and measurement of cell dry weight. One unit of inulinase activity was defined as the amount of enzyme catalyzing the liberation of 1 μmol of fructose min^-1 ^at pH 4.5 and 50°C. Specific enzyme activities are expressed per milligram of cell dry weight.

### RT-PCR analysis

Cells grown in YPD medium for 18 h were subsequently inoculated at 5% into YPD, YPSuc, YPRaf, YPInu, YPDSuc, YPDRaf or YPDInu, and after 4 h of incubation at 30°C or 45°C, total RNAs were isolated by the hot phenol method. RT-PCR analysis was performed as described previously (Lertwattanasakul et al. 2007; Sootsuwan et al. 2007). Primers used for *KmINU1*, *KmMIG1 *and *KmACT1 *were 5'-GTACAACCCAGCAGCCA-3' for KmINU1-213 and 5'-GCTTGGAGTCGGAGGAG-3' for KmINU1-784, 5'-CGGACGCATACTGGGGA-3' for KmMIG1-160 and 5'-ACCGAGTGGAGGGTTGT-3' for KmMIG1-707, and 5'-ACGTTGTTCCAATCTACGCC-3' for KmACT1-5 and 5'-AGAAGATG-GAGCCAAAGCAG-3' for KmACT1-3. Relative band intensities were determined using scanned images and UN-SCAN-IT software (Silk Scientific, Orem, UT, U.S.A.). Under our conditions, the RNA-selective RT-PCR was able to specifically detect mRNA because no band was observed when reverse transcriptase was omitted.

### Database search

Homology searching was performed by FASTA and BLAST in GenBank, NCBI, DDBJ, EMBL, and SWISS-PROT databases. Comparisons of nucleotide and amino acid sequences were conducted by Genetyx (Software Development, Tokyo). The *KmINU1 *sequence obtained from *K. marxianus *DMKU 3-1042 has been submitted to the DDBJ database under the accession number AB621573.

## Results

### Glucose effect on utilization of Suc, Raf or Inu

**Figure 1:**
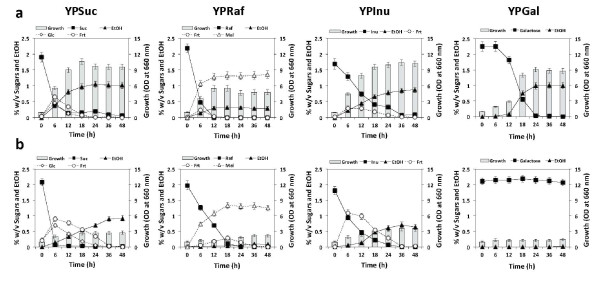
**Static batch fermentation of sucrose, raffinose or inulin in the absence of glucose**. Cells grown in YPD medium at 30°C for 18 h were inoculated into batch culture, which was conducted in 300-ml Erlenmeyer flask containing 100 ml of YP medium containing 2% glucose (YPD), sucrose (YPSuc), raffinose (YPRaf), inulin (YPInu) or galactose (YPGal) at 30°C (a) and 45°C (b) under a static condition as time indicated. Initial OD_660 _was adjusted to 1.0. Bars represent the ±SD for three independent experiments.

**Figure 2:**
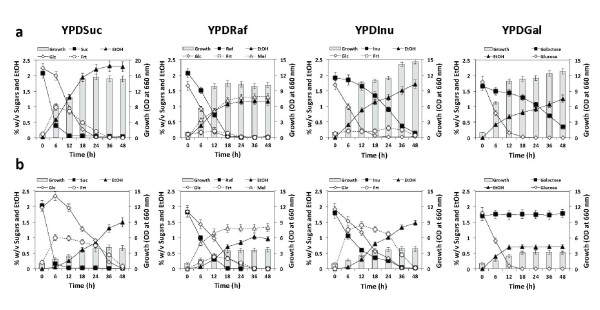
**Static batch fermentation of sucrose, raffinose or inulin in the presence of glucose**. Cells grown in YPD medium at 30°C for 18 h were inoculated into batch culture, which was conducted in 300-ml Erlenmeyer flask containing 100 ml of YP medium containing 2% sucrose (YPSuc), raffinose (YPRaf), inulin (YPInu) or galactose (YPGal) with 2% glucose at 30°C (a) and 45°C (b) under a static condition as time indicated. Initial OD_660 _was adjusted to 1.0. Bars represent the ±SD for three independent experiments.

To determine whether there is a glucose effect on utilization of Suc, Raf or Inu in *K. marxianus *DMKU 3-1042, its growth was compared on YPSuc, YPRaf and YPInu with or without Glc at 30°C or 45°C under a static condition (Figures 1 and 2; Tables 1 and 2). Growth on YPGal was also tested as a positive control for glucose repression.

**Table 1:** Parameters in YP medium containing a single sugar under a static condition at 30°C and 45°C

Medium	Temperature (°C)	**Max. *Y***_**p/s **_**(g/g)**	**Time of fermentation (h)**^**a**^	***μ***_**x/s **_**(h**^**-1**^**) at 6 h**	**Max. *μ***_**x/s **_**(h**^**-1**^**)**	***γ***_**s **_**(g/l h) at 6 h**	**Max. *γ***_**s **_**(g/l h)**
YPSuc	30	0.55 ± 0.04	24	0.77 ± 0.07	0.77 (6) ± 0.07	2.5 ± 0.02	2.5 (6) ± 0.02
	45	0.44 ± 0.05	48	0.18 ± 0.10	0.18 (6) ± 0.10	3.4 ± 0.03	3.4 (6) ± 0.03
YPRaf	30	0.14 ± 0.03	18	0.44 ± 0.04	0.44 (6) ± 0.04	2.9 ± 0.04	2.9 (6) ± 0.04
	45	0.04 ± 0.05	24	0.05 ± 0.09	0.06 (24) ± 0.10	1.2 ± 0.01	1.2 (6) ± 0.01
YPInu	30	0.52 ± 0.04	48	0.57 ± 0.11	0.57 (6) ± 0.11	0.66 ± 0.07	0.93 (12) ± 0.08
	45	0.58 ± 0.01	18	0.14 ± 0.08	0.14 (6) ± 0.08	1.4 ± 0.10	1.4 (6) ± 0.10
YPGal	30	0.45 ± 0.03	36	0.14 ± 0.06	0.85 (18) ± 0.12	0.0 ± 0.00	2.1 (18) ± 0.09
	45	-	-	-	-	-	-

Values in parenthesis represent cultivation times

*Max. Y*_*p/s *_maximum ethanol yield, *μ*_*x/s *_specific growth rate, *Max. μ*_*x/s *_maximum growth rate, *γ*_*s *_specific sugar utilization rate, *Max. γ*_*s *_maximum sugar utilization rate, ± SD from three independent experiments

^a ^Time required for the maximum ethanol concentration to be reached

**Table 2:** Parameters in YP medium containing mixed sugars with Glc under a static condition at 30°C and 45°C

Medium	Temperature (°C)	**Max. *Y***_**p/s **_**(g/g)**	**Time of fermentation (h)**^**a**^	***μ***_**x/s **_**(h**^**-1**^**) at 6 h**	**Max. *μ***_**x/s **_**(h**^**-1**^**)**	***γ***_**s **_**(g/l h) at 6 h**		**Max. *γ***_**s **_**(g/l h)**	
YPDSuc	30	0.53 ± 0.02	36	1.3 ± 0.05	1.3 (6) ± 0.05	Glc	0.42 ± 0.09	Glc	1.7 (12) ± 0.04
						Suc	2.8 ± 0.05	Suc	2.8 (6) ± 0.05
	45	0.37 ± 0.04	48	0.15 ± 0.08	0.19 (18) ± 0.05	Glc	-0.6 ± 0.03	Glc	1.1 (18) ± 0.03
						Suc	3.1 ± 0.12	Suc	3.1 (6) ± 0.12
YPDRaf	30	0.31 ± 0.05	36	0.78 ± 0.11	0.78 (6) ± 0.11	Glc	1.2 ± 0.10	Glc	1.3 (12) ± 0.05
						Raf	0.93 ± 0.08	Raf	1.3 (12) ± 0.01
	45	0.28 ± 0.01	36	0.14 ± 0.04	0.15 (12) ± 0.03	Glc	0.68 ± 0.09	Glc	1.0 (18) ± 0.02
						Raf	1.4 ± 0.10	Raf	1.4 (6) ± 0.10
YPDInu	30	0.48 ± 0.02	48	0.81 ± 0.09	0.81 (6) ± 0.09	Glc	1.2 ± 0.03	Glc	1.2 (12) ± 0.04
						Inu	0.11 ± 0.06	Inu	0.75 (24) ± 0.03
	45	0.40 ± 0.02	48	0.11 ± 0.03	0.16 (12) ± 0.01	Glc	0.63 ± 0.04	Glc	1.0 (18) ± 0.07
						Inu	1.2 ± 0.01	Inu	1.2 (6) ± 0.01
YPDGal	30	0.36 ± 0.04	48	0.96 ± 0.03	0.96 (6) ± 0.03	Glc	1.7 ± 0.05	Glc	1.7 (6) ± 0.05
						Gal	0.27 ± 0.03	Gal	0.29 (36) ± 0.02
	45	0.21 ± 0.03	18	0.13 ± 0.01	0.14 (12) ± 0.01	Glc	1.5 ± 0.06	Glc	1.5 (6) ± 0.06
						Gal	-	Gal	-

Values in parenthesis represent cultivation times

*Max. Y*_*p/s *_maximum ethanol yield, *μ*_*x/s *_specific growth rate, *Max. μ*_*x/s *_maximum growth rate, *γ*_*s *_specific sugar utilization rate, *Max. γ*_*s *_maximum sugar utilization rate, ±SD from three independent experiments

^a ^Time required for the maximum ethanol concentration to be reached

In the absence of Glc, Suc and Raf were rapidly consumed and were completely consumed within the first 12 h at 30°C, whereas Inu was consumed at a relatively slow rate (0.66 g/l h at 6 h) (Figure 1a; Table 1). The maximum growth level on Raf was low compared to that on the other two sugars because of the production of unmetabolizable melibiose. The rate of ethanol production on Suc was low at 45°C due to the slow uptake of Glc and Frt following hydrolysis of Suc (Figure 1b). The utilization of Gal was very slow with a delay of about 6 h compared to that under a shaking condition at 30°C (Rodrussamee et al. 2011) and hardly occurred at 45°C. The consumption of Suc and Inu at 45°C was slightly faster than that at 30°C and the consumption of Raf at 45°C was slower than that at 30°C (Figure 1b; Table 1).

The rate of Suc utilization in the presence of Glc was almost the same as that in the absence of Glc at both temperatures (Figure 2a, b; Table 2). The maximum ethanol yield from a mixture of Suc and Glc at 30°C was higher than that at 45°C, and the ethanol level was maintained until the end of the fermentation period examined. However, growth at 45°C was reduced to about 30% of that at 30°C. At 30°C, the rates of Raf and Inu utilization were reduced by 3 fold and 6 fold, respectively, in the presence of Glc (Table 2). Raf was consumed simultaneously with Glc, but the consumption of Inu was delayed after depletion of Glc. Both sugars were consumed much faster at 45°C than at 30°C. Almost no glucose repression was found in the utilization of Raf and Inu. This is presumably due to the availability of inulinase enzyme for hydrolytic reaction at a high temperature (see below). The effects of glucose repression on Gal and Raf utilization in *K. marxianus *DMKU 3-1042 were found to be significant but weaker than that and similar to that, respectively, in *S. cerevisiae *at 30°C (data not shown).

### Effect of 2-deoxyglucose (2-DOG) on utilization of Suc, Raf or Inu

**Figure 3:**
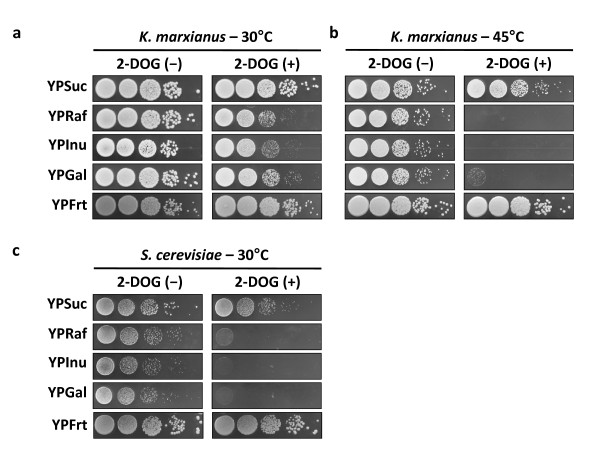
**Effect of 2-DOG on utilization of sucrose, raffinose or inulin**. Cells were grown in YPD medium to about 10^7 ^cells/ml, aliquots of 10-fold culture dilutions of cells were spotted onto agar plates containing YP medium supplemented with 2% fructose (YPFrt), 2% sucrose (YPSuc), 2% raffinose (YPRaf) or 2% inulin (YPInu) in the presence (+) or absence (-) of 0.01% 2-DOG, and the plates were incubated at 30°C (a) or 45°C (b) for 3 days. Galactose (Gal) was included as a positive control. *S. cerevisiae *was used as a reference strain for glucose repression (c).

To further examine the glucose effect on utilization of Suc, Raf and Inu, cell growth was compared on YPSuc, YPRaf and YPInu agar plates supplemented with 2-DOG as a glucose analogue at 30°C and 45°C (Figure 3a, b). At 30°C, growth was repressed by the addition of 2-DOG on Raf and Inu as on Gal, but almost no repression was observed on Suc. Interestingly, the extent of the repression was much weaker than that in *S. cerevisiae *at 30°C (Figure 3c). The repressive effect was more evident at 45°C. No growth was observed on Raf or Inu in the presence of 2-DOG at 45°C. This phenomenon is presumably due to the initial uptake of 2-DOG over Frt derived from Raf or Inu to the cells at the beginning of growth, hampering the uptake of Frt. On the other hand, *K. marxianus *could grow well even in the presence of 2-DOG when a high concentration of Frt was present at the early growth phase as in the case of YPFrt (Figure 3a, b).

**Figure 4:**
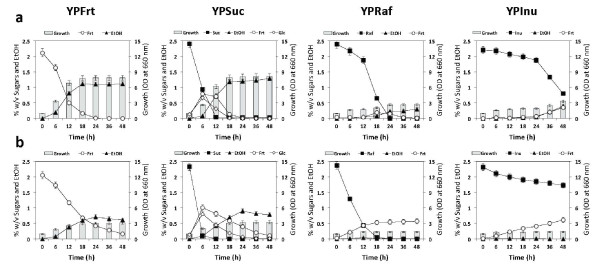
**Effect of 2-DOG on fructose uptake**. Cells grown in YPD medium at 30°C for 18 h were inoculated into sequential batch culture, which was conducted in 300-ml Erlenmeyer flask containing 100 ml of YP medium containing 2% fructose (YPFrt), 2% sucrose (YPSuc), 2% raffinose (YPRaf) or 2% inulin (YPInu) supplemented with 0.01% 2-DOG. Cultivation was continued further at 30°C (a) or 45°C (b) under a static condition as time indicated. Initial OD_660 _was adjusted to 1.0. Bars represent the ±SD for three independent experiments.

To determine the mechanism behind the phenomenon described above, we performed experiments in a liquid medium of YPFrt, YPSuc, YPRaf or YPInu supplemented with 2-DOG at 30°C and 45°C. The speed of Frt uptake in YPFrt at 45°C was found to be slower than that at 30°C (Figure 4). During the hydrolysis of Suc, Raf or Inu, Frt was accumulated at both temperatures and could be further utilized by the organism only at 30°C except for the case of YPSuc, where 2-DOG only slowed down the speed of Frt uptake at 45°C. However, the uptake of Frt was completely inhibited when cells were grown in YPRaf or YPInu at 45°C, and no cell growth was observed (Figure 4b). Considering the fact that Raf and Inu consumption was enhanced at 45°C when Glc was added together (Figure 2b), it is likely that 2-DOG was accumulated as 2-DOG-6-phosphate before hydrolysis of the sugars to prevent metabolic activities and cell growth. Taken together, the results obtained with 2-DOG for the consumption of the three sugars at 30°C were almost consistent with those obtained with Glc.

**Figure 5:**
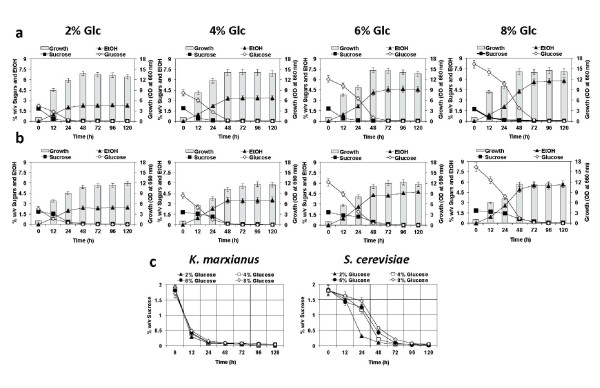
**Effect of glucose concentrations on hydrolysis of sucrose**. Cells grown in YPD medium at 30°C for 18 h were inoculated into sequential batch culture, which was conducted in 300-ml Erlenmeyer flask containing 100 ml of YP medium supplemented with a mixture of 2% sucrose (Suc), and various concentrations of glucose (Glc) as indicated. Cultivations of *K. marxianus *(a) and *S. cerevisiae *(b) were continued further at 30°C under a static condition as time indicated. The patterns of sucrose hydrolysis at different glucose concentrations were summarized (c). Initial OD_660 _was adjusted to 1.0. Bars represent the ±SD for three independent experiments.

The effect of extracellular Glc concentration on glucose repression in *S. cerevisiae *has been investigated, and it has been shown that the level of repression is correlated with increase in Glc concentration (Meijer et al. 1998). To further examine the effect of Glc concentration on utilization of Suc in *K. marxianus *DMKU 3-1042, we examined cell growth in 2% Suc supplemented with various concentrations of Glc under a static condition at 30°C (Figure 5). Unlike *S. cerevisiae*, increase in Glc concentration from 2-8% had almost no effect on the rate of Suc consumption in the yeast.

### Glucose effect on production and distribution of inulinase

**Figure 6:**
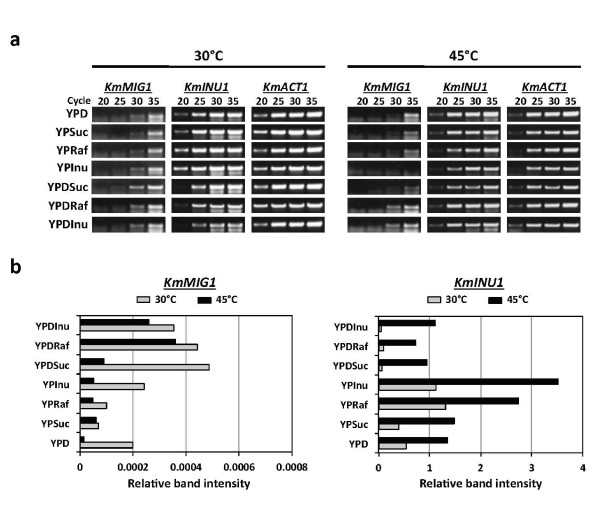
**Expression of *KmINU1 *in various sugars under a static condition**. Cells grown in YPD medium for 18 h were inoculated into YP supplemented with 2% glucose (YPD), sucrose (YPSuc), raffinose (YPRaf) or inulin (YPInu), or a mixture of these sugars with 2% glucose, and cultivated further for 4 h at 30°C or 45°C. Total RNA was then isolated and subjected to RT-PCR with primers specific to corresponding genes that amplify an approximately 500-bp DNA fragment. (a) After reverse transcriptase reaction, PCR products of 20, 25, 30 and 35 cycles were subjected to 0.9% agarose gel electrophoresis and stained with ethidium bromide. (b) Relative band intensities were determined using scanned image and UN-SCAN-IT software (Silk Scientific, Orem, UT, U.S.A.).

In order to examine the glucose effect on production or distribution of inulinase, with 6-h cultures in the liquid medium of YPSuc or YPInu in the presence or absence of Glc at 30°C and 45°C as described above, we measured inulinase activity in the supernatant and cell fractions and compared total activities under different conditions or activities of the two fractions (Table 3). In YPD, YPSuc and YPInu media, total inulinase activities were 600, 650 and 1920 U mg of cell dry weight^-1 ^at 30°C, respectively, and 1140, 1290 and 1390 U mg of cell dry weight^-1 ^at 45°C, respectively. The tendency in difference of these values was consistent with results of RT-PCR experiments (see Figure 6b) except for the case of YPInu, indicating that inulinase is induced by Inu but not by Suc at 30°C and by heat. In supernatant fractions, approximately 3-times higher inulinase activity was recovered at 45°C than that at 30°C in all media except for YPInu. The increase in total activity along with the temperature up-shift seems to reflect the increase in supernatant fraction activity, indicating facilitated secretion of inulinase at a high temperature.

**Table 3:** Production and distribution of inulinase in static batch cultures of *K. marxianus *DMKU 3-1042 in YP medium with 2% various carbon substrates

Medium	Temperature (°C)	**Total inulinase activity (U mg of cell dry wt**^**-1**^**)**^**a**^	**Inulinase activity (U mg of cell dry wt**^**-1**^**)**^**a**^	
			
			Supernatant (%)	Cell (%)
YPD	30	600	310 (52)	290 (48)
	45	1140	880 (77)	260 (23)
YPSuc	30	650	310 (48)	340 (52)
	45	1290	1000 (78)	290 (22)
YPInu	30	1920	1000 (52)	920 (48)
	45	1390	820 (59)	570 (41)
YPDSuc	30	640	410 (64)	230 (36)
	45	1570	1300(83)	270 (17)
YPDInu	30	590	320 (54)	270 (46)
	45	1230	960 (78)	270 (22)

^a ^Enzyme activities were measured with sucrose as substrate with samples taken at 6 h from static batch cultures as described in Materials and methods

Values are average from two independent experiments

Total inulinase activity in YPDInu was 3.3-times lower than that in YPInu at 30°C, but almost no such difference was observed between YPDSuc and YPSuc at both temperatures or between YPDInu and YPInu at 45°C. On the other hand, distribution of inulinase activity in supernatant fractions was hardly influenced by the addition of Glc.

### Alignments of conserved domains and upstream sequences of *K. marxianus *inulinase with those of glycoside hydrolase family 32 from other yeast species

*K. marxianus *DMKU 3-1042 possesses only one copy of *KmINU1 *encoding for inulinase as a counterpart of *ScSUC2 *encoding for invertase in *S. cerevisiae *(The genome sequence will be published elsewhere.). The two enzymes belong to the glycoside hydrolase family 32 (GH32) group in carbohydrate-degrading enzymes. Comparison of primary sequences deduced from nucleotide sequences revealed that KmInu1p bears several motifs of WMNDPNG (block A), WHLY(F/Y)Q (block B), WGHA(T/V)S (block B1), FSGSMV(V/I) (block C), FRDPKVF (block D), QYECPGL (block E) and I(I/L)ELY (block G), which are conserved among invertases and inulinases in yeast GH32 enzymes (Additional file 1). The conserved domains of KmInu1p in DMKU 3-1042 showed sequence identity of 100% to those of the corresponding enzyme from the other strains of *K. marxianus*. The enzyme is classified into exoinulinase on the basis of the presence of Asp in block A, whereas endoinulinase has Glu at the Asp position. The carboxyl groups of Asp in block A and Glu in block E are involved in the catalytic activity of ß-fructofuranosidases (Reddy et al. 1996). The Glu residue in block E may act as a proton donor in the catalytic reaction, as reported for invertase from *S. cerevisiae *(Reddy et al. 1996). The Asp residue in block D, which is conserved in all inulinases, is related to substrate recognition (Nagem et al. 2004).

However, there are conflicting data on the regulation of inulinase production among different strains of *K. marxianus*. We thus compared the upstream non-coding sequence of *KmINU1 *in DMKU 3-1042 with those of the corresponding genes in four other *K marxianus *strains, CBS 6556, ATCC 12424, Y1 and CBS 834 (Nucleotide sequences of *KmINU1 *of CBS 4857 and IW 9801 are not available.). The inulinase gene in CBS 6556 has been reported to be repressed by Glc (Rouwenhorst et al. 1988), but there is no available information on the regulation of *KmINU1 *genes in other strains. Approximately 700-bp upstream sequences of *KmINU1 *from the five strains were aligned, and two putative Mig1 elements (consensus sequence, WWWWTSYGGGG) were found in all strains (Additional file 2). These findings and evidence based on disruption of the *KmMIG1 *gene (Cassart 1997) suggest that the negative regulation of *KmINU1 *is dependent on KmMig1p, a key effector for glucose repression as in strain CBS 6556. However, we could not compare such upstream sequences with other strains due to the lack of nucleotide sequences of *KmINU1 *in databases, of which UCD (FST) 55-82 has been claimed to be free from glucose repression (Parekh and Margaritis 1985).

### Glucose effect on expression of *KmINU1*

To determine whether the regulation of inulinase by Glc occurs at the transcriptional level, RT-PCR was carried out with total RNA from cells grown for 4 h at 30°C or 45°C under the same condition as that for other experiments by liquid culture (Figure 6). The band intensities of *KmMIG1 *and *KmINU1 *were converted to relative values by comparison with that of *KmACT1 *as an internal control. The values thus reflect the expression level of each gene tested. The expression profiles indicated that *KmINU1 *was similarly expressed in Glc and Suc and its expression level was increased about 2-3 times in Raf and Inu at 30°C. Notably, the expression level of *KmINU1 *at 45°C was more than 2-times higher than that at 30°C in all media tested. In the presence of Glc, the expression level was greatly reduced in Suc, Raf and Inu at both temperatures except for a slight reduction in Suc at 45°C. On the other hand, the expression level of *KmMIG1 *was increased by the addition of glucose and was reduced at 45°C compared to that at 30°C. These expression alterations were oppositely consistent with those of *KmINU1*. Therefore, these results suggest that *KmINU1 *is inducible by Raf or Inu and negatively controlled by Glc via KmMig1p in strain DMKU 3-1042.

## Discussion

Results presented in this paper showed the utilization capability of Suc, Raf and Inu at a high temperature in *K. marxianus *DMKU 3-1042, which is the most thermotolerant among strains available (Nonklang et al. 2008) and efficiently utilizes hexose and pentose sugars (Rodrussamee et al. 2011), as well as the glucose effects on consumption of these sugars and on the expression of *KmINU1 *for inulinase responsible for their hydrolysis. This work thus also provides an insight into the fundamental mechanism of glucose repression in *K. marxianus*. The strain can assimilate the three sugars at a high temperature even under a static condition, though the respiratory yeast exhibits a sugar assimilation activity much higher under a shaking condition than that under a static condition (Rodrussamee et al. 2011). The hydrolysis and consumption of Suc, Raf or Inu in the presence of Glc were found to be preferable at a high temperature, and no detectable effect of glucose repression on Suc consumption was observed. Therefore, this strain is applicable for high-temperature ethanol fermentation with a biomass such as sugar cane juice containing mainly Suc, Glc and Frt.

Although the same inulinase is involved in the hydrolysis of Suc, Raf and Inu in *K. marxianus *DMKU 3-1042, an effect of glucose repression was observed on the consumption of Raf and Inu but not on that of Suc at the low temperature (Figures 1 and 2). The effect of sugar-specific glucose repression on consumption of sugars was consistent with that on production and secretion of inulinase, which was evaluated on the basis of inulinase activity (Table 3). Inconsistent results, however, were obtained by transcript analysis, revealing that *KmINU1 *was down-regulated in the presence of Glc in all media tested. Coincidentally, the repression of *KmINU1 *was oppositely proportional to the expressional alteration of *KmMIG1 *by Glc. At the high temperature, however, no further effect of glucose repression on the consumption of Raf and Inu and on the production and secretion of inulinase was observed. Rather, the rise of temperature positively affected both production and distribution of inulinase to efficiently degrade these sugars in the strain. These results allow us to speculate that the increased inulinase production apparently overcomes the reduction in transcript by the glucose effect. Similarly, for the phenomenon that glucose repression of the expression of *KmINU1 *in YPSuc has no effect on Suc consumption, it is possible that the amount of inulinase produced under the condition of glucose repression is sufficient for cells to consume Suc as efficiently as that under the Glc-free condition.

An effect of glucose repression was observed on the utilization capability of Suc in *S. cerevisiae *but not in *K. marxianus*. The localization of Suc-hydrolyzing enzymes, invertase and inulinase in *S. cerevisiae *and *K. marxianus*, respectively, may be different or altered by cultivation conditions. In *S. cerevisiae*, almost all invertase molecules produced are retained inside the cell wall. On the other hand, a large proportion of inulinase molecules in *K. marxianus *are secreted into the culture medium (Nam et al., 1993). In contrast to invertase, inulinase is able to hydrolyze fructans such as inulin and levan (Kushi et al. 2000). These polysaccharides, however, are too large to enter the cell wall, and thus their hydrolysis occurs outside the cell wall (Rouwenhorst et al. 1990). We noticed that inulinase activity in the supernatant fraction at 45°C was approximately 3-times higher than that at 30°C under conditions with or without Glc except for YPInu, but the activity in the cell fraction was not altered (Table 3). The rise of activity in the supernatant fraction reflects the increase in *KmINU1 *expression and also indicates an increase in secretion of inulinase into the culture medium. Therefore, a high temperature condition facilitates inulinase release into the culture medium presumably by change in cell wall structure as previously proposed (Kushi et al. 2000; Rouwenhorst et al. 1988).

This study has further clarified useful characteristics of *K. marxianus *DMKU 3-1042 for fermentation. First, the elevation of temperature stimulates production of inulinase. This may amplify the reactivity of inulinase since the enzyme is relatively heat-resistant with optimum temperature around 50°C and 70°C for Inu and Suc as substrates, respectively (Rouwenhorst et al. 1988). Second, the elevation of temperature enhances the secretion of inulinase. Third, the consumption of these sugars is less sensitive to glucose repression. These characteristics encourage us to apply the thermotolerant yeast for high-temperature ethanol fermentation with biomass containing these sugars with Glc.

Although there are conflicting reports on the regulation of utilization of Inu among *K. marxianus *strains (Grootwassink and Fleming 1980; Grootwassink and Hewitt 1983; Parekh and Margaritis 1985; Rouwenhorst et al. 1988; Cruz-Guerrero et al. 1995; Schwan et al. 1997), our analyses revealed that the primary structure of inulinase including functional domains in different strains is highly conserved and that their inulinase genes share conserved upstream sequences including two possible Mig1 elements. Therefore, we think that the conflicting results regarding the regulation of Inu utilization are mainly due to differences in experimental conditions, including temperature, which alter the localization or activity of inulinase.

## List of abbreviations

YP: yeast extract and peptone; Glc: glucose; Suc: sucrose; Raf: raffinose; Inu: inulin; Frt: fructose; Mel: melibiose; Gal: galactose; 2-DOG: 2-deoxyglucose; GH32: glycoside hydrolase family 32; SD: standard deviation; RT-PCR: reverse transcriptase-polymerase chain reaction; NITE: National Institute of Technology and Evaluation; NBRC: NITE Biological Resource Center

## Competing interests

The authors declare that they have no competing interests.

## Supplementary Material

Additional file 1 **Alignment of proteins of the glycoside hydrolase family 32 (GH32) subfamilies from yeast species**. Species are abbreviated by the following: Deb.hansenii = *Debaryomyces hansenii*, Schw.occidentalis = *Schwanniomyces occidentalis*, Pic.anomala = *Pichia anomala*, Pic.jadinii = *Pichia jadinii*, Can.guilliermondii = *Candida guilliermondii*, S.cerevisiae = *Saccharomyces cerevisiae*, S.monacensis = *Saccharomyces monacensis*, S.pastorianus = *Saccharomyces pastorianus*, S.bayanus = *Saccharomyces bayanus*, S.cariocanus = *Saccharomyces cariocanus*, Y.lipolytica = *Yarrowia lipolytica*, S.paradoxus = *Saccharomyces paradoxus*, Ash.gossypii = *Ashbya gossypii*, Vand.polyspora = *Vanderwaltozyma polyspora*, Km = *Kluyveromyces marxianus*, Kluy.lactis = *Kluyveromyces lactis*, Zygo.rouxii = *Zygosaccharomyces rouxii*, Schiz.pombe = *Schizosaccharomyces pombe*. Conserved residues are shaded by different intensities based on conservation level in the alignment. Residues in black show 100% conservation, residues in dark grey show ≥75% conservation, and residues in light grey show ≥50% conservation. Asterisks indicate residues previously confirmed or suspected to be part of the active site (Reddy et al. 1996). The eight conserved motifs (A, B, B1, C, D, E, F and G) are indicated at the top. Click here for file

Additional file 2 **Alignment of upstream sequences of inulinase genes from various strains of *K. marxianus***. Km = *Kluyveromyces marxianus*. Strains names are indicated after Km-. Asterisks indicate conserved nucleotides. The putative binding sites of KmMig1p are shaded. Click here for file
