# Supplementary material for: Utilization capability of sucrose, raffinose and inulin and its less-sensitiveness to glucose repression in thermotolerant yeast Kluyveromyces marxianus DMKU 3-1042
Source: AMB Express. 2011 Jul 19;1:20. doi: 10.1186/2191-0855-1-20 (PMC3222316; doi:10.1186/2191-0855-1-20)
Supplement: Additional file 1 — Alignment of proteins of the glycoside hydrolase family 32 (GH32) subfamilies from yeast species. Species are abbreviated by the following: Deb.hansenii = Debaryomyces hansenii, Schw.occidentalis = Schwanniomyces occidentalis, Pic.anomala = Pichia anomala, Pic.jadinii = Pichia jadinii, Can.guilliermondii = Candida guilliermondii, S.cerevisiae = Saccharomyces cerevisiae, S.monacensis = Saccharomyces monacensis, S.pastorianus = Saccharomyces pastorianus, S.bayanus = Saccharomyces bayanus, S.cariocanus = Saccharomyces cariocanus, Y.lipolytica = Yarrowia lipolytica, S.paradoxus = Saccharomyces paradoxus, Ash.gossypii = Ashbya gossypii, Vand.polyspora = Vanderwaltozyma polyspora, Km = Kluyveromyces marxianus, Kluy.lactis = Kluyveromyces lactis, Zygo.rouxii = Zygosaccharomyces rouxii, Schiz.pombe = Schizosaccharomyces pombe. Conserved residues are shaded by different intensities based on conservation level in the alignment. Residues in black show 100% conservation, residues in dark grey show ≥75% conservation, and residues in light grey show ≥50% conservation. Asterisks indicate residues previously confirmed or suspected to be part of the active site (Reddy et al. 1996). The eight conserved motifs (A, B, B1, C, D, E, F and G) are indicated at the top. [file 2191-0855-1-20-S1.PDF]

```
Deb.hansenii            ------------------------------------------------------------
Schw.occidentalis       ------------------------------------------------------------
Pic.anomala             ------------------------------------------------------------
Pic.jadinii             ------------------------------------------------------------
Can.guilliermondii      ------------------------------------------------------------
S.cerevisiae            ------------------------------------------------------------
S.monacensis            ------------------------------------------------------------
S.pastorianus           ------------------------------------------------------------
S.bayanus               ------------------------------------------------------------
S.cariocanus            ------------------------------------------------------------
Y.lipolytica            -------------------------MKLATAFTILTAV---------------------- 13
S.paradoxus             ------------------------------------------------------------
Ash.gossypii            ---------------------------------MYFSK---------------------- 5
Vand.polyspora-1        MKVTIESFVCFVSLYVSLVSSAAIELPTFTIPSYTIPSYTIPSYTIPSYTIPSYTIPSYT 60
Vand.polyspora-2        -----------------MRTSSVAKSATLVAASLSLAN---------------------- 21
Km-DMKU3-1042           ------------------------------------------------------------
Km-CBS6556              ------------------------------------------------------------
Km-CBS4857              ------------------------------------------------------------
Km-ATCC12424            ------------------------------------------------------------
Km-Y1                   ------------------------------------------------------------
Km-CBS834               ------------------------------------------------------------
Km-IW9801               ------------------------------------------------------------
Kluy.lactis             ------------------------------------------------------------
Zygo.rouxii             ------------------------------------------------------------
Schiz.pombe-1           -----------------------------------------------------------M 1
Schiz.pombe-2           ------------------------------------------------------------

Deb.hansenii            -----MKITTLVASILMSVLLEP-----------VIASFIDTGKDTSA------------ 32
Schw.occidentalis       ----MVQVLSVLVIPLLTLFFGY-----------VASSSIDLSVDTSE------------ 33
Pic.anomala             ----MIQLSPLLLLPLFSVFNSI-----------ADAS--------TE------------ 25
Pic.jadinii             -----MSLTKDASEDQEDIKSLT-----------MNTSLVD-----SS------------ 27
Can.guilliermondii      --------MRAFLALIFLTFVMN-----------VESS---------------------- 19
S.cerevisiae            -----------MLLQAFIFLLAG-----------FAAKISALMTNETS------------ 26
S.monacensis            -----------MLLQAFIFLLAG-----------FAAKISALMTNETS------------ 26
S.pastorianus           -----------MLLQAFIFLLAG-----------FAAKISALMTNETS------------ 26
S.bayanus               -----------MLLQAFIFLLAG-----------FAAKISALMTNETS------------ 26
S.cariocanus            -----------MLLQAFIFLLAG-----------FAAKISALMTNETS------------ 26
Y.lipolytica            -----LAAPLAAPAEAFLFLLAG-----------FAAKISASMTNETS------------ 45
S.paradoxus             -----------MLLQAFIFLLAG-----------FAAKISALMTNETS------------ 26
Ash.gossypii            -----LLSAVALCSSASALYVPGDADVNVQNKDQTPTALPEEPLNGTA------------ 48
Vand.polyspora-1        IPSYTIPSYTIPSYTIPSYSIPSYSIPSYSIPSYTIPSFTTLTFSYETSIPIPSI----- 115
Vand.polyspora-2        ----AASSVTTTTLTANSASVTPF-VGSDITTTWPSTIFSDLYTSVDN------------ 64
Km-DMKU3-1042           -----MKLAYSLLLPLAGVS---------ASVINYKRDGDSKAITNTT------------ 34
Km-CBS6556              -----MKLAYSLLLPLAGVS---------ASVINYKRDGDSKAITNTT------------ 34
Km-CBS4857              -----MKLAYSLLLPLAGVS---------ASVINYKRDGDSKAITNTT------------ 34
Km-ATCC12424            -----MKFAYSLLLPLAGVS---------ASVINYKRDGDSKAITNTT------------ 34
Km-Y1                   -----MKFAYSLLLPLAGVS---------ASVINYKRDGDSKAITNTT------------ 34
Km-CBS834               -----MKFAYSLLLPLAGVS---------ASVINYKRDGDSKAITNTT------------ 34
Km-IW9801               ------------------------------------MDGDSKAITNTT------------ 12
Kluy.lactis             -----MLKLLSLMVPLASAAVIHR---RDANISAIASEWNSTSNSSSS------------ 40
Zygo.rouxii             -----------------------------------------MGLQD-------------- 5
Schiz.pombe-1           FLKYILASGICLVSLLSSTNAAPRHLYVKRYPVIYNASNITEVSNSTTVPPPPFVNTTAP 61
Schiz.pombe-2           ------------------------------------------------------------

                                                           A               B
Deb.hansenii            -------------------YNRPLIHLTPNVGWLNDPNGLFYDKKTSVWHAYYQYNPND- 72
Schw.occidentalis       -------------------YNRPLIHFTPEKGWMNDPNGLFYDKTAKLWHLYFQYNPNA- 73
Pic.anomala             -------------------YLRPQIHLTPDQGWMNDPNGMFYDRKDKLWHVYFQHNPDKK 66
Pic.jadinii             -------------------IYRPLVHLTPPVGWMNDPNGLFYDSSESTYHVYYQYNPND- 67
Can.guilliermondii      ---------------------RPLMAFTPSHGWMNDPNGQFYDSKNELWHLYYQYNPND- 57
S.cerevisiae            --------------------DRPLVHFTPNKGWMNDPNGLWYDAKEGKWHLYFQYNPND- 65
S.monacensis            --------------------DRPLVHFTPNKGWMNDPNGLWYDAKEGKWHLYFQYNPND- 65
S.pastorianus           --------------------DRPLVHFTPNKGWMNDPNGLWYDAKEGKWHLYFQYNPND- 65
S.bayanus               --------------------DRPLVHFTPNKGWMNDPNGLWYDAKEGKWHLYFQYNPND- 65
S.cariocanus            --------------------DRPLVHFTPNKGWMNDPNGLWYDEKDAKWHLYFQYNPND- 65
Y.lipolytica            --------------------DRPLVHFTPNKGWMNDPNGLWYDEKDAKWHLYFQYNPND- 84
S.paradoxus             --------------------DRPLVHFTPNKGWMNDPNGLWYDEKDAKWHLYFQYNPND- 65
Ash.gossypii            --------------------YRPIMHATPSQGWMNDPNGLWYDTKEEVYHVYYQYNPAD- 87
Vand.polyspora-1        --------PFTVSSFNTTQDNKPLIHATPPEGWMNDPNGLWYDSKDELYHMYYQYNPND- 166
Vand.polyspora-2        ------------ETYGGFEESRPLIHATPPKGWMNDPNGLWYDASEELYHMYYQYSPNE- 111
Km-DMKU3-1042           -----------------FSLNRPSVHFTPSHGWMNDPNGLWYDAKEEDWHLYYQYNPAA- 76
Km-CBS6556              -----------------FSLNRPSVHFTPSHGWMNDPNGLWYDAKEEDWHLYYQYNPAA- 76
Km-CBS4857              -----------------FSLNRPSVHFTPSHGWMNDPNGLWYDAKEEDWHLYYQYNPAA- 76
Km-ATCC12424            -----------------FSLNRPSVHFTPSHGWMNDPNGLWYDAKEEDWHLYYQYNPAA- 76
Km-Y1                   -----------------FSLNRPSVHFTPSHGWMNDPNGLWYDAKEEDWHLYYQYNPAA- 76
Km-CBS834               -----------------FSLNRPSVHFTPSHGWMNDPNGLWYDAKEEDWHLYYQYNPAA- 76
Km-IW9801               -----------------FSLNRPSVHFTPSHGWMNDPNGLWYDAKEEDWHLYYQYNPAA- 54
Kluy.lactis             -----------------LSLNRPAVHYSPEEGWMNDPNGLWYDAKEEDWHIYYQYYPDA- 82
Zygo.rouxii             -------------------LEHPLVHLTPPHGWLNDPNGLWYDAKDELWHCYFQHNPDS- 45
Schiz.pombe-1           NGTCLGNYNEYLPSGYYNATDRPKIHFTPSSGFMNDPNGLVYT--GGVYHMFFQYSPKT- 118
Schiz.pombe-2           -----------------MPVRPCIHFTPPEGFMNDPNGLVYS--NGKWHLFFQWNPTG- 39
                                                           *
```

Additional file 1

```
                              B1                        C
Deb.hansenii            TIWGQPLYWGHSSSKDLTHWEEHQVALGPQNDDEG----IFSGSIVIDYNNTSGFFDE-- 126
Schw.occidentalis       TAWGQPLYWGHATSNDLVHWDEHEIAIGPEHDNEG----IFSGSVVVDHNNTSGFFNS-- 127
Pic.anomala             SIWATPVTWGHSTSKDLLTWDYHGNALEPENDDEG----IFSGSVVVDRNNTSGFFND-- 120
Pic.jadinii             TIWGLPLYWGHATSDDLLTWDHHAPAIGPENDDEG----IYSGSIVIDYDNTSGFFDD-- 121
Can.guilliermondii      TVWGTPLYWGHATSKDLSTWKDYGATIGPDRDEDG----IFSGNIVVDHNNTSGFFND-- 111
S.cerevisiae            TVWGLPLFWGHATSNDLTHWQDEPVAIAPKRNDSG----AYSGSMVIDHNNTSGFFND-- 119
S.monacensis            TVWGLPLFWGHATSNDLTHWQDEPVAIAPKRNDSG----AYSGSMVIDHNNTSGFFND-- 119
S.pastorianus           TVWGLPLFWGHATSNDLTHWQDEPVAIAPKRNDSG----AYSGSMVIDHNNTSGFFND-- 119
S.bayanus               TVWGLPLFWGHATSNDLTHWQDEPVAIAPKRNDSG----AYSGSMVIDHNNTSGFFND-- 119
S.cariocanus            TVWGTPLFWGHATSDDLTHWQDEPIAIAPKRNDSG----AFSGSMVVDYNNTSGFFND-- 119
Y.lipolytica            TVWGTPLFWGHATSDDLTNWEDQPIAIAPKRNDSG----AFSGSMVVDYNNTSGFFND-- 138
S.paradoxus             TVWGTPLYWGHATSDDLTHWQDEPIAIAPKRNDSG----AFSGSMVVDYNNTSGFFND-- 119
Ash.gossypii            TVWGVPLYWGHLTSKDLQSWEDHGVAIRPPRNDSG----AFSGSAVVDTNNTSGFFND-- 141
Vand.polyspora-1        TVWALPIVWGHKTSKNLTIWDDAGIAMAPTDTITG----FYSGSVVVDYNNTSGFFNS-- 220
Vand.polyspora-2        TYWSLPIVWGHKTSKNLTVWDDAGIAMAPTDNTTG----FYSGSVVVDYNNTSGFFNS-- 165
Km-DMKU3-1042           TIWGTPLYWGHAVSKDLTSWTDYGASLGPGSDDAG----AFSGSMVIDYNNTSGFFNS-- 130
Km-CBS6556              TIWGTPLYWGHAVSKDLTSWTDYGASLGPGSDDAG----AFSGSMVIDYNNTSGFFNS-- 130
Km-CBS4857              TIWGTPLYWGHAVSKDLTSWTDYGASLGPGSDDAG----AFSGSMVIDYNNTSGFFNS-- 130
Km-ATCC12424            TIWGTPLYWGHAVSKDLTSWTDYGASLGPGSDDAG----AFSGSMVIDYNNTSGFFNS-- 130
Km-Y1                   TIWGTPLYWGHAVSKDLTSWTDYGASLGPGSDDAG----AFSGSMVIDYNNTSGFFNS-- 130
Km-CBS834               TIWGTPLYWGHAVSKDLTSWTDYGASLGPGSDDAG----AFSGSMVIDYNNTSGFFNS-- 130
Km-IW9801               TIWGTPLYWGHAVSKDLTSWTDYGASLGPGSDDAG----AFSGSMVIDYNNTSGFFNS-- 108
Kluy.lactis             PHWGLPLTWGHAVSKDLTVWDEQGVAFGPEFETAG----AFSGSMVIDYNNTSGFFNS-- 136
Zygo.rouxii             TVWKQPICWGHSVSKDAIIWDYKGLAIVPPDSDGG----VFSGSVVVDNRNTSELFDE-- 99
Schiz.pombe-1           -LTAGEVHWGHTVSKDLIHWENYPIAIYPDEHENGVLSLPFSGSAVVDVHNSSGLFSN-- 175
Schiz.pombe-2           -NQAGNQHWGHAVSKNLYKWKLLPTALAPGDDHG----LMFSGSAVIDKTNSSGFFESGF 94

                                                                           D
Deb.hansenii            ----SIDKDQRVVAIY-TNSIPDTQTQDIAYSLDGGETFTKYKKNPVIDVNSTQFRDPKV 181
Schw.occidentalis       ----SIDPNQRIVAIY-TNNIPDLQTQDIAFSLDGGYTFTKYENNPVIDVSSNQFRDPKV 182
Pic.anomala             ----STDPEQRIVAIY-TNN-AQLQTQEIAYSLDKGYSFIKYDQNPVINVNSSQQRDPKV 174
Pic.jadinii             ----STRPEQRIVAIY-TNNLPDVETQDIAYSTDGGYTFEKYENNPVIDVNSTQFRDPKV 176
Can.guilliermondii      ----SIDPRQRVVAIY-TYNTEGSQTQHVAYSLDGGYTFEKYEHNPVLDVDNINFRDPKV 166
S.cerevisiae            ----TVDPRQRCVAIW-TYNTPESEEQYISYSLDGGYTFTEYQKNPVLAANSTQFRDPKV 174
S.monacensis            ----TVDPRQRCVAIW-TYNTPESEEQYISYSLDGGYTFTEYQKNPVLAANSTQFRDPKV 174
S.pastorianus           ----TVDPRQRCVAIW-TYNTPESEEQYISYSLDGGYTFTEYQKNPVLAANSTQFRDPKV 174
S.bayanus               ----TVDPRQRCVAIW-TYNTPESEEQYISYSLDGGYTFTEYQKNPVLAANSTQFRDPKV 174
S.cariocanus            ----TIDPRQRCVAIW-TYNTPESEEQYISYSLDGGYTFTEYQKNPVLAANSTQFRDPKV 174
Y.lipolytica            ----TIDPRQRCVAIW-TYNTPESEEQYISYSLDGGYTFTEYQKNPVLAANSTQFRDPKV 193
S.paradoxus             ----TIDPRQRCVAIW-TYNTPESEEQYISYSLDGGYTFTEYQKNPVLAANSTQFRDPKV 174
Ash.gossypii            ----SIDPAQRVVAIW-TYNTPESETQWISYSLDGGYTFIDYANNPVLDLNSTQFRDPKV 196
Vand.polyspora-1        ----STDPRQRAVAIY-TYNTPEAEVQCVAYSLDGGYSFIQYESNPVLSNNSTQFRDPKV 275
Vand.polyspora-2        ----TTDPRQRAVAIF-TYNTPEAEVQCVAYSLDGGYTFIQYESNPVLSNNSTQFRDPKV 220
Km-DMKU3-1042           ----SVDPRQRAVAVW-TLSKGPSQAQHISYSLDGGYTFQHYSDNAVLDINSSNFRDPKV 185
Km-CBS6556              ----SVDPRQRAVAVW-TLSKGPSQAQHISYSLDGGYTFQHYSDNAVLDINSSNFRDPKV 185
Km-CBS4857              ----SVDPRQRAVAVW-TLSKGPSQAQHISYSLDGGYTFQHYSDNAVLDINSSNFRDPKV 185
Km-ATCC12424            ----SVDPRQRAVAVW-TLSKGPSQAQHISYSLDGGYTFEHYTDNAVLDINSSNFRDPKV 185
Km-Y1                   ----SVDPRQRAVAVW-TLSKGPSQAQHISYSLDGGYTFEHYTDNAVLDINSSNFRDPKV 185
Km-CBS834               ----SVDPRQRAVAVW-TLSKGPSQAQHISYSLDGGYTFEHYTDNAVLDINSSNFRDPKV 185
Km-IW9801               ----SVDPRQRAVAVW-TLSKGPSQAQHISYSLDGGYTFEHYTDNAVLDINSSNFRDPKV 163
Kluy.lactis             ----STDPRQRVVAIW-TLDYSGSETQQLSYSHDGGYTFTEYSDNPVLDIDSDAFRDPKV 191
Zygo.rouxii             ----STDPRQRVVAIW-TQDADGIQRQMISYSMDGGYSFKDYAHNPVLDINNSNFRDPKV 154
Schiz.pombe-1           ----DTIPEERIVLIYTDHWTGVAERQAIAYTTDGGYTFKKYSGNPVLDINSLQFRDPKV 231
Schiz.pombe-2           FSRKSVDPEERIVLIYTTHYD-NRETQNIAYSLDGGITFIKYKKNPILDIKESQFRDPKV 153

                                                                        E *
Deb.hansenii            FWHE--ETN---KWIMVVSKSQEYKIQIFGSLDLKTWDLHSNFTS-GYLGNQYECPGLIK 235
Schw.occidentalis       FWHE--RFK---SMDHGCSEIARVKIQIFGSANLKNWVLNSNFSS-GYYGNQYGMSRLIE 236
Pic.anomala             LWHD--ESN---QWIMVVAKTQEFKVQIYGSPDLKKWDLKSNFTSNGYLGFQYECPGLFK 229
Pic.jadinii             IWYE--ETE---QWVMTVAKSQEYKIQIYTSDNLKDWSLASNFSTKGYVGYQYECPGLFE 231
Can.guilliermondii      FWHE--PTN---QWIMVIALSQQFKIQIYGSIDLTNWSLHSNFTG-GLFGFQYECPGLIE 220
S.cerevisiae            FWYE--PSQ---KWIMTAAKSQDYKIEIYSSDDLKSWKLESAFANEGFLGYQYECPGLIE 229
S.monacensis            FWYE--PSQ---KWIMTAAKSQDYKIEIYSSDDLKSWKLESAFANEGFLGYQYECPGLIE 229
S.pastorianus           FWYE--PSQ---KWIMTAAKSQDYKIEIYSSDDLKSWKLESAFANEGFLGYQYECPGLIE 229
S.bayanus               FWYE--PSQ---KWIMTAAKSQDYKIEIYSSDDLKSWKLESAFANEGFLGYQYECPGLIE 229
S.cariocanus            FWYE--PSQ---KWIMTAAKSQDYKIEIYSSDDLKSWKLESAFANEGFLGYQYECPGLIE 229
Y.lipolytica            FWYE--PSQ---KWIMTAAKSQDYKIEIYSSDDLKSWKLESAFANEGFLGYQYECPGLIE 248
S.paradoxus             FWYE--PSQ---KWIMTAAKSQEYKIEIFSSDDLKSWKLESAFANEGFLGYQYECPGLIE 229
Ash.gossypii            IWHE--ESQ---KWIMTVVLSHKYAIQIYSSDNLREWTLESEFKNHGLLGFQYECPGLAK 251
Vand.polyspora-1        IWHE--ESQ---KWIMTVAKTQEYKVAIYSSSDLKDWTLESEVEKVGVLGYQYECPGLAK 330
Vand.polyspora-2        IWHE--ESQ---KWIMTIAKTQEYKVSIYSSSDLKDWTLESEVEKVGVLGYQYECPGLAR 275
Km-DMKU3-1042           FWHEGENGE-DGRWIMAVAESQVFSVLFYSSPNLKNWTLESNFTHHGWTGTQYECPGLVK 244
Km-CBS6556              FWHEGENGE-DGRWIMAVAESQVFSVLFYSSPNLKNWTLESNFTHHGWTGTQYECPGLVK 244
Km-CBS4857              FWHEGENGE-DGRWIMAVAESQVFSVLFYSSPNLKNWTLESNFTITVWTGTQYECPGLVK 244
Km-ATCC12424            FWHEGENGE-DGRWIMAVAESQVFSVLFYSSPNLKNWTLESNFTHHGWTGTQYECPGLVK 244
Km-Y1                   FWHEGENGE-DGRWIMAVAESQVFSVLFYSSPNLKNWTLESNFTHHGWTGTQYECPGLVK 244
Km-CBS834               FWHEGENGE-DGRWIMAVAESQVFSVLFYSSPNLKNWTLESNFTHHGWTGTQYECPGLVK 244
Km-IW9801               FWHEGENGE-DGRWIMAVAESQVFSCLFYSSPNLKNWTLESNFTHHGWTGTQYECPGLVK 222
Kluy.lactis             FWYQGEDSESEGNWVMTVAEADRFSVLIYSSPDLKNWTLESNFSREGYLGYNYECPGLVK 251
Zygo.rouxii             IWHK--ETG---RWIMVVALSQKFEISIYSSKDLIHWQYESGFAVRGFKGLQYECPGLIK 209
Schiz.pombe-1           IWDF--DAN---RWVMIVAMSQNYGIAFYSSYDLIHWTELSVFSTSGYLGLQYECPGMAR 286
Schiz.pombe-2           FWHE--ESR---AWIMVVVLAQKYKVLFYHSLNLRDWVKLSEFGSAGVLGYQYECPDFVR 208
                                                                     **
```

Additional file 1 (continued)

```
Deb.hansenii           VPIEN----------TNDYK---------------------------------------- 245
Schw.occidentalis      VPIEN----------SDKSK---------------------------------------- 246
Pic.anomala            LPIENPL------NDTVTSK---------------------------------------- 243
Pic.jadinii            ATIENPK------SGDPEKK---------------------------------------- 245
Can.guilliermondii     VPVEG----------TDELK---------------------------------------- 230
S.cerevisiae           VPT----------EQDP------------------------------------------- 236
S.monacensis           VPT----------EQDP------------------------------------------- 236
S.pastorianus          VPT----------EQDP------------------------------------------- 236
S.bayanus              VPT----------EQDP------------------------------------------- 236
S.cariocanus           VPT----------EQDP------------------------------------------- 236
Y.lipolytica           VPT----------EQDP------------------------------------------- 255
S.paradoxus            VPT----------EQDA------------------------------------------- 236
Ash.gossypii           IPV----------SKPANCE------MQLKDVSYPVKN---------------------- 273
Vand.polyspora-1       ISL----------PDVVLESGSNNKTIPNSSSSK-------------------------- 354
Vand.polyspora-2       ISL----------PNVTLASGSSNMTVPYPSTYSNGTA---------------------- 303
Km-DMKU3-1042          VPY----------DSVADSSS----NSSDSKPDS-------------------------- 264
Km-CBS6556             VPY----------DSVADSSS----NSSDSKPDS-------------------------- 264
Km-CBS4857             VPY----------DSVADSS-----NSSDSKPDS-------------------------- 263
Km-ATCC12424           VPY----------DSVVDSS-----NSSDSKPDS-------------------------- 263
Km-Y1                  VPY----------DSVVDSS-----NSSDSKPDS-------------------------- 263
Km-CBS834              VPY----------DSVVDSS-----NSSDSKPDS-------------------------- 263
Km-IW9801              VPY----------DSVVDSS-----NSSDSKPDS-------------------------- 241
Kluy.lactis            VPYVKNTTYASAPGSNITSSGPLHPNSTVSFSNSSSIAWNASSVPLNITLSNSTLVDETS 311
Zygo.rouxii            VPLLDAN------GQEVKDT---------------------------------------- 223
Schiz.pombe-1          VPVEG----------TDEYK---------------------------------------- 296
Schiz.pombe-2          LPIEG----------TDEFR---------------------------------------- 218

                                                                          F
Deb.hansenii           --------WVMFLAINPGSPA-GGSSNQYFIGEFDGFEFKQDDSITRVMDAGKDFYAFQT 296
Schw.occidentalis      --------WVMFLAINPGSPL-GGSINQYFVGDFDGFQFVPDDSQTRFVDIGKDFYAFQT 297
Pic.anomala            --------WVLLLAINPGSPL-GGSINEYFIGDFDGTTFHPDDGATRFMDIGKDFYAFQS 294
Pic.jadinii            --------WVMVLAINPGSPL-GGSINEYFVGDFNGTEFIPDDDATRFMDTGKDFYAFQA 296
Can.guilliermondii     --------WVMFIAINPGLPL-GGSSNQYFIGSFDGFEFVPDDSQARLMDYGKDFYAFQT 281
S.cerevisiae           ----SKSHWVMFISINPGAPA-GGSFNQYFVGSFNGTHFEAFDNQSRVVDFGKDYYALQT 291
S.monacensis           ----SKSHWVMFISINPGAPA-GGSFNQYFVGSFNGTHFEAFDNQSRVVDFGKDYYALQT 291
S.pastorianus          ----SKSHWVMFISINPGAPA-GGSFNQYFVGSFNGTHFEAFDNQSRVVDFGKDYYALQT 291
S.bayanus              ----SKSHWVMFISINPGAPA-GGSFNQYFVGSFNGTHFEAFDNQSRVVDFGKDYYALQT 291
S.cariocanus           ----SKSYWVMFISINPGAPA-GGSFNQYFVGSFNGTHFEAFDNQSRVVDFGKDYYALQT 291
Y.lipolytica           ----SKSYWVMFISINPGAPA-GGSFNQYFVGSFNGTHFEAFDNQSRVVDFGKDYYALQT 310
S.paradoxus            ----SKSYWVMFISINPGAPA-GGSFNQYFVGSFNGTHFEAFDNQSRVVDFGKDYYALQT 291
Ash.gossypii           ---NTDYVWVMFLAINPGGPQ-GGNFNQYFIGDFDGKKFTPFSEQTRFLDHGKDFYAFQG 329
Vand.polyspora-1       ------DAWVLFISINPGAPQ-GGSYVEYFIGDFNGTVFTPFSRETQALDDGKDYYAFQT 407
Vand.polyspora-2       ---TPTDAWVMFISVNPGAPN-GGSFVQYFIGDFNGTTFTPFTEQTQTLDDGEDYYALQT 359
Km-DMKU3-1042          -------AWVLFVSINPGGPL-GGSVTQYFVGDFNGTHFTPIDDQTRFLDMGKDYYALQT 316
Km-CBS6556             -------AWVLFVSINPGGPL-GGSVTQYFVGDFNGTHFTPIDDQTRFLDMGKDYYALQT 316
Km-CBS4857             -------AWVLFVSINPGGPL-GGSVTQYFVGDFNGTHFTPIDDQTRFLDMGKDYYALQT 315
Km-ATCC12424           -------AWVLFVSINPGGPL-GGSVTQYFVGDFNGTHFTPIDGQTRFLDMGKDYYALQT 315
Km-Y1                  -------AWVLFVSINPGGPL-GGSVTQYFVGDFNGTHFTPIDGQTRFLDMGKDYYALQT 315
Km-CBS834              -------AWVLFVSINPGGPL-GGSVTQYFVGDFNGTHFTPIDGQTRFLDMGKDYYALQT 315
Km-IW9801              -------AWVLFVSINPGGPL-GGSVTQYFVGDFNGTHFTPIDGQTRFLDMGKDYYALQT 293
Kluy.lactis            QLEEVGYAWVMIVSFNPGSIL-GGSGTEYFIGDFNGTHFEPLDKQTRFLDLGKDYYALQT 370
Zygo.rouxii            -----KDNWVLYISINPGAPQ-GGSATEYFIGEFDGKVFQPRDNQVRLMDLGKDFYAFQT 277
Schiz.pombe-1          --------WVLFISINPGAPL-GGSVVQYFVGDWNGTNFVPDDGQTRFVDLGKDFYASAL 347
Schiz.pombe-2          --------WVLIVSINPGSSINGGSMVQYFIGDFDGQTFTPIDSASRILDCGHDCYATQT 270

Deb.hansenii           FS---DNEQDVIGLAWASNWQYANVVP-TNPWRSSMSLARKYTLG-YVNQNVETKIMTLI 351
Schw.occidentalis      FS---EVEHGVLGLAWASNWQYADQVP-TNPWRSSTSLARNYTLR-YVIQ--MLKLTANI 350
Pic.anomala            FDNT-EPEDGALGLAWASNWQYANTVP-TENWRSSMSLVRNYTLK-YVDVNPENYGLTLI 351
Pic.jadinii            FFN--APENRSIGVAWSSNWQYSNQVPDPDGYRSSMSSIREYTLR-YVSTNPESEQLILC 353
Can.guilliermondii     FDNA-PKELGVVGLAWASNWQYANLAP-TKEWRSSMTLARQMTLA-SRNMNPETKVLSLL 338
S.cerevisiae           FFNTDPTYGSALGIAWASNWEYSAFVP-TNPWRSSMSLVRKFSLNTEYQANPETELINLK 350
S.monacensis           FFNTDPTYGSALGIAWASNWEYSAFVP-TNPWRSSMSLVRKFSLNTEYQANPETELINLK 350
S.pastorianus          FFNTDPTYGSALGIAWASNWEYSAFVP-TNPWRSSMSLVRKFSLNTEYQANPETELINLK 350
S.bayanus              FFNTDPTYGSALGIAWASNWEYSAFVP-TNPWRSSMSLVRKFSLNTEYQANPETELINLK 350
S.cariocanus           FFNTDPTYGSALGIAWASNWEYSAFVP-TNPWRSSMSLVRKFSLDTEYQANPETELINLK 350
Y.lipolytica           FFNTDPTYGSALGIAWASNWEYSAFVP-TNPWRSSMSLVRKFSLNTEYQANPETELINLK 369
S.paradoxus            FFNTDPTYGSALGIAWASNWEYSAFVP-TNPWRSSMSLVRKFSLNTEYQANPETELINLK 350
Ash.gossypii           FYNSQFKD-SFLGIAWASNWQYSAYVP-TNPWRSSMSLARKLTVR-PYNPTPESVQLVLN 386
Vand.polyspora-1       FFNS--PDNSTLGVAWASNWQYGQYVP-TYPWRSSMSLVRNLTLE-YFQANPESKILKLK 463
Vand.polyspora-2       FFNS--ADNSTLGVAWASNWKYAQSVD-TYPWKSSMSLVRKFTLD-YFQANPESKILSLK 415
Km-DMKU3-1042          FFNTP-NEKDVYGIAWASNWQYAQQAP-TDPWRSSMSLVRQFTLK-DFSTNPNSADVVLN 373
Km-CBS6556             FFNTP-NEKDVYGIAWASNWQYAQQAP-TDPWRSSMSLVRQFTLK-DFSTNPNSADVVLN 373
Km-CBS4857             FFNTP-NEKDVYGIAWASNWQYAQQAP-TDPWRSSMSLVRQFTLK-DFSTNPNSADVVLN 372
Km-ATCC12424           FFNTP-NEKDVYGIAWASNWQYAQQAP-TDPWRSSMSLVRQFTLK-DFSTNPNSADVVLN 372
Km-Y1                  FFNTP-NEKDVYGIAWASNWQYAQQAP-TDPWRSSMSLVRQFTLK-DFSTNPNSADVVLN 372
Km-CBS834              FFNTP-NEKDVYGIAWASNWQYAQQAP-TDPWRSSMSLVRQFTLK-DFSTNPNSADVVLN 372
Km-IW9801              FFNTP-NEKDVYGIAWASNWQYAQQAP-TDPWRSSMSLVRQFTLK-DFSTNPNSADVVLN 350
Kluy.lactis            FFNTP-NEVDVLGIAWASNWQYANQVP-TDPWRSSMSLVRNFTIT-EYNINSNTTALVLN 427
Zygo.rouxii            FYNTP-NDKDVIGMAWASNWQYTNQTP-TSQYRSCLTMLRKLHLQ-KLQITPEYSEINLF 334
Schiz.pombe-1          YHS-SSANADVIGVGWASNWQYTNQAPT-QVFRSAMTVARKFTLR-DVPQNPMTNLTSLI 404
Schiz.pombe-2          FG--NAPDGRVISISWASNWNYTNDVPMRMKHRGMFTIPRELTLC-YTHLNQETRGLVLR 327
```

Additional file 1 (continued)

```
Deb.hansenii          QTPILNN-----------LDVINKVEKNNHLLTKNDSVITNFSSS---TGLLDFNTTFKV 397
Schw.occidentalis     DKSVLPDS----------INVVDKLKKKNVKLTNKKPIKTNFKGS---TGLFDFNITFKV 397
Pic.anomala           QKPVYDTKETRLNETLKTLETINEYEVNDLKLDKSSFVATDFNTERNATGVFEFDLKFTQ 411
Pic.jadinii           QKPFFVNET--------DLKVVEEYKVSNSSLTVDHTFGSSFANSN-TTGLLDFNMTFTV 404
Can.guilliermondii    QKPIFGES------------VVAANKISKRNITGQDEQAVKIHKNS--TGTFLFDITFSV 384
S.cerevisiae          AEPILNISN---------AGPWLHFAS---NSTLTKANSFSVDLSNS-TGTLEFELVYAV 397
S.monacensis          AEPILNISN---------AGPWLHFAS---NSTLTKANSFSVDLSNS-TGTLEFELVYAV 397
S.pastorianus         AEPILNISN---------AGPWLHFAS---NSTLTKANSFSVDLSNS-TGTLEFELVYAV 397
S.bayanus             AEPILNISN---------AGPWLHFAS---NSTLTKANSFSVDLSNS-TGTLEFELVYAV 397
S.cariocanus          AEPILNISN---------AGPWSRFAT---NTTLTKANSYNVDLSNS-TGTLEFELVYAV 397
Y.lipolytica          AEPILNISN---------AGPWSRFAT---NTTLTKANSYNVDLSNS-TGTLEFELVYAV 416
S.paradoxus           AEPILNVSN---------AGPWSRFAT---NTTLTKSNSYSVDLSNS-TGSLEFELVYAV 397
Ash.gossypii          SEPVFVPED---------MEFNSNFSSWK-DLKLTSGKEEVFEFGSTPLGAFEFNLTFTA 436
Vand.polyspora-1      SQPVIDYDC---------FTSNSDVIKFS-NLSSLSTLDSIYSFSNSSEGILEFNLTWSV 513
Vand.polyspora-2      SEPCVDYDV---------FDLNAGTLYTL-NNATDDFMHAKITT-NSSQGLLEFNMTWSV 464
Km-DMKU3-1042         SQPVLNYDA---------LRKNGTTYSIT-NYTVTSENGKKIKLDNP-SGSLEFHLEYVF 422
Km-CBS6556            SQPVLNYDA---------LRKNGTTYSIT-NYTVTSENGKKIKLDNP-SGSLEFHLEYVF 422
Km-CBS4857            SQPVLNYDA---------LRKNGTTYSIT-NYTVTSENGKKIKLDNP-SGSLEFHLEYVF 421
Km-ATCC12424          SQPVLNYDA---------LRKNGTTYSIT-NYTVTSENGKKIKLDNP-SGSLEFHLEYVF 421
Km-Y1                 SQPVLNYDA---------LRKNGTTYSIT-NYTVTSENGKKIKLDNP-SGSLEFHLEYVF 421
Km-CBS834             SQPVLNYDA---------LRKNGTTYSIT-IYTVTSENGKKIKLDNP-SGSLEFHLEYVF 421
Km-IW9801             SQPVLNYDA---------LRKNGTTYSIT-NYTVTSENGKKIKLDNP-SGSLEFHLEYVF 399
Kluy.lactis           SQPVLDFTS---------LRKNGTSYTLE-NLTLNSSSHEVLEFEDP-TGVFEFSLEYSV 476
Zygo.rouxii           SAPLWDQDS---------LVNLAPPKSLTPDAPLLPNHGLNINLDQG-EGLLEFTWEWSV 384
Schiz.pombe-1         QTPLNVSLLR-------------DETLFTAPVINSSSSLSGSPITLPSNTAFEFNVTLSI 451
Schiz.pombe-2         QRPVNLHHLY-------------YPDSLPAPLLLN--RVCEFPTVWTSATVFYLAVSIPK 372

Deb.hansenii          VGESIDSN-SLSNIEILIHSQMSNSSTESIKVGFDRSVSAFYFNRD-IPNVEFNNNPYFT 455
Schw.occidentalis     LNLNVSP--GKTHFDILINSQELNSSVDSIKIGFDSSQSLFYIDRH-IPNVEFPRKQFFT 454
Pic.anomala           TDLKMGYSNMTTQFGLYIHSQTVKGSQETLQLVFDTLSTTWYIDRT-TQHSFQRNSPVFT 470
Pic.jadinii           NGTTDVTQKDSVTFELRIKS---NQSDEAIALGYDYNNEQFYINRA-TESYFQRTNQFFQ 460
Can.guilliermondii    DLLKNQTG------QLQVIS---GQNGESIRAGFDPTAGQFFVDRG-NTSGLK-ENPFFT 433
S.cerevisiae          NTTQSVSKSVFSDLSLWFKGL--EDPEEYLRMGFEASASSFFLDRGNSKVKFVKENPYFT 455
S.monacensis          NTTQSVSKSVFSDLSLWFKGL--EDPEEYLRMGFEASASSFFLDRGNSKVKFVKENPYFT 455
S.pastorianus         NTTQSVSKSVFSDLSLWFKGL--EDPEEYLRMGFEASASSFFLDRGNSKVKFVKENPYFT 455
S.bayanus             NTTQSVSKSVFSDLSLWFKGL--EDPEEYLRMGFEASASSFFLDRGNSKVKFVKESPYFT 455
S.cariocanus          NTTQTISKSVFADLSLWFKGL--EDPEEYLRMGFEVSASSFFLDRGNSKVKFVKENPYFT 455
Y.lipolytica          NTTQTISKSVFADLSLWFKGL--EDPEEYLRMGFEVSASSFFLDRGNSKVKFVKENPYFT 474
S.paradoxus           NSTKTVSKSVFADLSLWFKGL--EDPEEYLRMGFEVSASSFFLDRGNSKVKFVKENPYFT 455
Ash.gossypii          NDTG-LSKHSLGDFSIYLEGA--KDPDEYLRLGYSTQAADFFFDRGNSKVSFVRENPFFT 493
Vand.polyspora-1      NSSS-YDNHDFADLSLKLRGG--LNPFEYLRLGYLANVNSFFIDRGHSTNNWVNENPFYT 570
Vand.polyspora-2      NASA-YDNSEAADLSLYLRGN--QFDDEYLWLGYIANAGAFYLDRGNTGSPFTATCPLFN 521
Km-DMKU3-1042         NGSPDIKSNVFADLSLYFKGN--NDDNEYLRLGYETNGGAFFLDRGHTKIPFVKENLFFN 480
Km-CBS6556            NGSPDIKSNVFADLSLYFKGN--NDDNEYLRLGYETNGGAFFLDRGHTKIPFVKENLFFN 480
Km-CBS4857            NGSPDIKSNVFADLSLYFKGN--NDDNEYLRLGYETNGGAFFLDRGHTKIPFVKENLFFN 479
Km-ATCC12424          NGSPDIKSNVFADLSLYFKGN--NDDNEYLRLGYETNGGAFFLDRGHTKIPFVKENLFFT 479
Km-Y1                 NGSPDIKSNVFADLSLYFKGN--NDDNEYLRLGYETNGGAFFLDRGHTKIPFVKENLFFT 479
Km-CBS834             NGSPDIKSNVFADLSLYFKGN--NDDNEYLRLGYETNGGAFFLDRGHTKIPFVKENLFFT 479
Km-IW9801             NGSPDIKSNVFADLSLYFKGN--NDDNEYLRLGYETNGGAFFLDRGHTKIPFVKENLFFT 457
Kluy.lactis           N-FTGIHNWVFTDLSLYFQGD--KDSDEYLRLGYEANSKQFFLDRGHSNIPFVQENPFFT 533
Zygo.rouxii           QDKL-VPKADFCGITMFLKDQ--ETSNQFLSLGFEANSSAFFLDRGNTGSHFTETNPLFT 441
Schiz.pombe-1         NYTEGCTTGYCLGRIIIDSDD--PYRLQSISVDVDFAASTLVINRAKAQMGWF--NSLFT 507
Schiz.pombe-2         SIVLESPMEFLCLTWSTTPDV--ETSKEYFELGYRFHDGAVYVERGVCSSSWK--YPLYP 428

                                                        G
Deb.hansenii          NKFSTYVEPSHYDEDDMPVYKIYGIVDKNIIELYFNDGTQTMTNTFFMSEDKYPHQIEIA 515
Schw.occidentalis     DKLAAYLEPLDYDQD-LRVFSLYGIVDKNIIELYFNDGTVAMTNTFFMGEGKYPHDIQIV 513
Pic.anomala           ERISTYVEKIDTTDQ-GNVYTLYGVVDRNIIELYFNDGSIAMTNTFFFREGKIPTSFEVV 529
Pic.jadinii           ERWSTYVQPLTITESGDKQYQLYGLVDNNIIELYFNDGAFTSTNTFFLEKGK-PSNVDIV 519
Can.guilliermondii    DKTSAYVEPWKHQND-LPVYKMFGVIDGNLIEVFLNDGIATLTNTFFIPGTEGLEYLEIE 492
S.cerevisiae          NRMSVNNQPFKSEND-LSYYKVYGLLDQNIIELYFNDGDVVSTNTYFMTTGNALGSVNMT 514
S.monacensis          NRMSVNNQPFKSEND-LSYYKVYGLLDQNIIELYFNDGDVVSTNTYFMTTGNALGSVNMT 514
S.pastorianus         NRMSVNNQPFKSEND-LSYYKVYGLLDQNIIELYFNDGDVVSTNTYFMTTGNALGSVNMT 514
S.bayanus             NRMSVNNQPFKSEND-LSYYKVYGLLDQNIIELYFNDGDVVSTNTYFMTTGNALGSVNMT 514
S.cariocanus          NRMSVNNQPFKTEDD-LSYYKVYGLLDQNIIELYFNDGDVVSTNTYFMTTGNSLGSVNMT 514
Y.lipolytica          NRMSVNNQPFKSEND-LSYYKVYGLLDQNIIELYFNDGDVVSTNTYFMTTGNALGSVNMT 533
S.paradoxus           NRMSLNNQPFKTEDD-LSYYKVYGLLDQNIIELYFNDGDVVSTNTYFMTTGNSLGSVNMT 514
Ash.gossypii          NKMAINMEPWEILAPGVKVFKVRAIFDVDLIELFFNEGTAASTNTYFLTEENHPASLKFK 553
Vand.polyspora-1      NKLSVNLQPFDYVNDEISTYKVYGIIDRNIMELYFNDGFQVSTNTFFFGDYNYISSVELS 630
Vand.polyspora-2      TRTAVNVQPCEYVSDEITTYKVRGIVDRNIIELYFNDGAQVSTNTFFFMDGDYIETCELK 581
Km-DMKU3-1042         HQLAVTNPVSNYTTN---VFDVYGVIDKNIIELYFDNGNVVSTNTFFFSTNNVIGEIDIK 537
Km-CBS6556            HQLAVTNPVSNYTTN---VFDVYGVIDKNIIELYFDNGNVVSTNTFFFSTNNVIGEIDIK 537
Km-CBS4857            HQLAVTNPVSNYTTN---VFDVYGVIDKNIIELYFDNGNVVSTNTFFFSTNNVIGEIDIK 536
Km-ATCC12424          HQLAVTNPVSNYTTN---VFDVYGVIDKNIIELYFDNGNVVSTNTFFFSTNNVIGEIDIK 536
Km-Y1                 HQLAVTNPVSNYTTN---VFDVYGVIDKNIIELYFDNGNVVSTNTFFFSTNNVIGEIDIK 536
Km-CBS834             HQLAVTNPVSNYTTN---VFDVYGVIDKNIIELYFDNGNVVSTNTFFFSTNNVIGEIDIK 536
Km-IW9801             HQLAVTNPVSNYTTN---VFDVYGVIDKNIIELYFDNGNVVSTNTFFFSTNNVIGEIDIK 514
Kluy.lactis           QRLSVSNPPSSNSS----TFDVYGIVDRNIIELYFNNGTVTSTNTFFFSTGNNIGSIIVK 589
Zygo.rouxii           RNLSADLTPYRTFQG-TSIYKVHGILDRNIIELYFNDGALAATYTFFYLENRPIRWVHME 500
Schiz.pombe-1         PSFANDIY--IYGN-----VTLYGIVDNGLLELYVNNGEKTYTNDFFFLQGATPGQISFA 560
Schiz.pombe-2         ERCTSSVPPSSYEDN--YILEIEAVVDHSIIEVYLQGGIMCLTNAYYFKGDEPLQYYYLR 486
```

Additional file 1 (continued)

```
Deb.hansenii            SNVDG-QFELQSLLIRELNN------- 534
Schw.occidentalis       TDTEEPLFELESVIIRELNK------- 533
Pic.anomala             CDSEKSFITIDELSVRELARK------ 550
Pic.jadinii             ASSSK------EAYHRGPAD------- 533
Can.guilliermondii      SSSDAIHIVELEVKELKLRATS----- 514
S.cerevisiae            TGVDN-LFYIDKFQVREVK-------- 532
S.monacensis            TGVDN-LFYIDKFQVREVK-------- 532
S.pastorianus           TGVDN-LFYIDKFQVREVK-------- 532
S.bayanus               TGVDN-LFYIDKFQVREVK-------- 532
S.cariocanus            ---------------------------
Y.lipolytica            TGVDN-LFYIDKFQVREVK-------- 551
S.paradoxus             TGVDD-LFYIDKFQVREVK-------- 532
Ash.gossypii            TSVDN-VFTVNELSLRQLTF------- 572
Vand.polyspora-1        VGKDG-VFDIQEFSVRQLHLK------ 650
Vand.polyspora-2        VNKMG-VFSIDEFSFRQLALKK----- 602
Km-DMKU3-1042           SPYDK-AYTINSFNVTQFNV------- 556
Km-CBS6556              SPYDK-AYTINSFNVTQFNV------- 556
Km-CBS4857              SPYDK-AYTINSFNVTQFNL------- 555
Km-ATCC12424            SPYDK-AYTINSFNVTQFNV------- 555
Km-Y1                   SPYDK-AYTINSFNVTQFNV------- 555
Km-CBS834               SPYDK-AYTINSFNVTQFNV------- 555
Km-IW9801               SPYDN-PYTINSFNVTQFNV------- 533
Kluy.lactis             SGVDD-VYEIESLKVNQFYVD------ 609
Zygo.rouxii             SSVND-VFKIKELRFTELAIKSPSKTY 526
Schiz.pombe-1           AFQGVSFNNVTVTPLKTIWNC------ 581
Schiz.pombe-2           VPTGASLAKSGMQPLLNNRPHS----- 508
```

Additional file 1 (continued)
