# Supplementary material for: Utilization capability of sucrose, raffinose and inulin and its less-sensitiveness to glucose repression in thermotolerant yeast Kluyveromyces marxianus DMKU 3-1042
Source: AMB Express. 2011 Jul 19;1:20. doi: 10.1186/2191-0855-1-20 (PMC3222316; doi:10.1186/2191-0855-1-20)
Supplement: Additional file 2 — Alignment of upstream sequences of inulinase genes from various strains of K. marxianus. Km = Kluyveromyces marxianus. Strains names are indicated after Km-. Asterisks indicate conserved nucleotides. The putative binding sites of KmMig1p are shaded. [file 2191-0855-1-20-S2.PDF]

```
Km-Y1          -TACCCAGGTATCCGGTTGTAGTTGGCACTGGGGATGGAAAAAAAA-GATTGAGATTGAT -652
Km-ATCC12424   ----CCAGGTATCCGGTTGTAGTTGGCACTGGGGATGGAAAAAAAAAGATTGAGATTGAT -654
Km-CBS834      ---CCCAGGTATCCGGTTGTAGTTGGCACTGGGGATGGAAAAAAAAAGATTGAGATTGAT -653
Km-DMKU3-1042  -----------TCCGGTTGTAGTTGGCACTGGGGATGGAAAAAAA-----TGATGTTGAT -666
Km-CBS6556     TACCCCAGGTATCCGGTTGTAGTTGGCACTGGGGATGGAAAAAAA-----TGATGTTGAT -655
                          **********************************     ***  *****

Km-Y1          GTTGAGTTAGTTGGCTTGGGTTGAGTTGAGTCAATTAGGGCGTGAAAGTATCACCACTTT -592
Km-ATCC12424   GTTGAGTTAGTTGGCTTGGGTTGAGTTGAGTCAATTAGGGCGTGAAAGTATCACCACTTT -594
Km-CBS834      GTTGAGTTAGTTGGCTTGGGTTGAGTTGAGTCAATTAGGGCGTGAAAGTATCACCACTTT -593
Km-DMKU3-1042  GTTGAGTTAGTTG-----GGTTGAGTTGAGTCAATTAGTGCGTGAAAGTATCACCACTTT -611
Km-CBS6556     GTTGAGTTAGTTG-----GGTT-----GAGTCAATTAGTGCGTGAAAGTATCACCACTTT -605
               *************     ****     *********** *********************

Km-Y1          TGTCATCCGGCGTTTCTGTGGGAATCACATA----CACACGCACAATTCATTGGAGCGCT -536
Km-ATCC12424   TGTCATCCGGCGTTTCTGTGGGAATCACATA----CACACGCACAATTCATTGGAGCGCT -538
Km-CBS834      TGTCATCCGGCGTTTCTGTGGGAATCACATA----CACACGCACAATTCATTGGAGCGCT -537
Km-DMKU3-1042  TGTCATCCGGCGTTTCTGTGGGAATCAT-------CACACACACAATTTATTGGAGCGCT -558
Km-CBS6556     TGTCATCCGGCGTTTCTGTGCGAATCACACACACACACACACACAGTTTATTGGAGCGCT -545
               ******************** ******        ***** **** ** ***********

Km-Y1          TGTTTTTGGCGAATTCGTAATTGTTCTGCGGTGCAGTTCTGTGTGCATTTTTCCTGGGGT -476
Km-ATCC12424   TGTTTCTGGCGAATTCGTAATTGTTCTGCGGTGCAGTTCTGTGTGCATTTTTCCTGGGGT -478
Km-CBS834      TGTTTCTGGCGAATTCGTAATTGTTCTGCGGTGCAGTTCTGTGTGCATTTTTCCTGGGGT -477
Km-DMKU3-1042  TGTTTCTGGCGAATTCGTAATTGTTCTGCGGTGCGGTTCTGTGTGCATTTTTCCTGGGGT -498
Km-CBS6556     TGTTTCTGGCGTATTCGTAATTGTTCTGCGGTGCGGTTCTGTGTGCATTTTTCCTGGGGT -485
               ***** ***** ********************** *************************

Km-Y1          GTCTGCCGCACCTACCCATCACCCACGCCGTGGGTTTGAGCCATGGCGGAGGTACGACTG -416
Km-ATCC12424   GTCTGCCGCACCTACCCATCACCCACGCCGTGGGTTTGAGCCATGGCGGAGGTACGACTG -418
Km-CBS834      GTCTGCCGCACCTACCCATCACC-ACGCCGTGGGTTTGAGCCATGGCGGAGGTACGACTG -418
Km-DMKU3-1042  GTCTGCCGCACCTACTCATCACCCACGCCGTGGGTTTGAGCCATGGCGGAGGTACGACTG -438
Km-CBS6556     GTCTGCCGCACCTACTCATCACCCACGCCGTGGGTTTGAGCCATGGCGGAGGTACGACTG -425
               *************** ******* ************************************

Km-Y1          ACCGACTGGCTGCCTGTCTGC------------CTGGCTGACTGACAGCAGGAAAAGAGG -368
Km-ATCC12424   ACCGACTGGCTGCCTGTCTGC------------CTGGCTGACTGACAGCAGGAAAAGAGG -370
Km-CBS834      ACCGACTGGCTGCCTGTCTGC------------CTGGCTGACTGACAGCAGGAAAAGAGG -370
Km-DMKU3-1042  ACTGGCTGCCTGCCTGCCTGCCTGCCTGCCTGACTGACTGCCTGACTGCAGGAAAAGAGG -378
Km-CBS6556     ACTGGCTGCCTGCCTGCCTG------------ACTGACTGCCTGACTGCAGGAAAAGAGG -377
               ** * *** ******* ***             *** *** ***** *************

Km-Y1          GTTTCCAGGGAAAAACTTTTCCTGTGTTAATCCGGCCGTGCGCCGCTGCTCCAAAATCCA -308
Km-ATCC12424   GTTTCCAAGGAAAAACTTTTCCTGTGTTAATCCGGCCGTGCGCCGCTGCTCCAAAATCCA -310
Km-CBS834      GTTTCCAAGGAAAAACTTTTCCTGTGTTAATCCGGCCGTGCGCCGCTGCTCCAAAATCCA -310
Km-DMKU3-1042  GTTTCGAAGGAAAAACTTTTCCTGTGTTAATCCGGCCGTGCGCCGCTGCTCCAAAATCCA -318
Km-CBS6556     GTTTCGAAGGAAAAACTTTTCCTGTGTTAATCCGGCCGTGCGCCGCTGCTCCAAAATCCA -317
               ***** * ****************************************************

Km-Y1          TCTTCATGAGAAGGAGTTTGAAAAAA------AATTCACATATAAAAGGCGTATCTCGAG -254
Km-ATCC12424   TCTTCATGAGAAGGAGTTTGAAAAAA------AATTCACATATAAAAGGCGTATCTCGAG -256
Km-CBS834      TCTTCATGAGAAGGAGTTTGAAAAAA------AATTCACATATAAAAGGCGTATCTCGAG -256
Km-DMKU3-1042  TCTTCATGAGAAGGAGTTTGAAAAAACAAAAAAATTCACATATAAAAAGCGTATCTCGAG -258
Km-CBS6556     TCTTCATGAGAAGGAGTTTGAAAAAACAAAAAAATTCACATATAAAAAGCGTATCTCGAG -257
               **************************      *************** ************

Km-Y1          ATCTCAAGGTCTCCCTTGAATGAAGTTTGCCAATTGTAACTCATCCTTTATTCTTATTCT -194
Km-ATCC12424   ATCTCAAAGTCTCCCTTGAATGAAGTTTGCCAATTGTAACTCATCCTTTATTCTTATTCT -196
Km-CBS834      ATCTCAAAGTCTCCCTTGAATGAAGTTTGCCAATTGTAACTCATCCTTTATTCTTATTCT -196
Km-DMKU3-1042  ATCTCAAAGTCTCCCTTGAATCGTGTTTGCCAGTTGTAACTCATCCTTTATTCTTCTATT -198
Km-CBS6556     ATCTCAAAGTCTCCCTTGAATCGTGTTTGCCAGTTGTAACTCATCCTTTATTCTTCTATT -197
               ******* *************   ******** ********************** *  *

Km-Y1          CTCTCTCTCTCTTCTCTTCCCTTAATTAGCAATTAAATCCGGGGTAAGGAAGAATTACTA -134
Km-ATCC12424   CTCTCTCTCTCTTCTCTTCCCCTAATTAGCAATTAAATCCGGGGTAAGGAAGAATTACTA -136
Km-CBS834      CTCTCTCTC--TTCTCTTCCCCTAATTAGCAATTAAATCCGGGGTAAGGAAGAATTACTA -138
Km-DMKU3-1042  CTATCTCTCTCTTTCCTTCCCCTAATCAGCAATTAAATCCGGGGTAAGGAAGAATTACTA -138
Km-CBS6556     CTATCTCTCTCTTTCCTTCCCCTAATCAGCAATTAAATCCGGGGTAAGGAAGAATTACTA -137
               ** ******  **  ****** **** *********************************
```

Additional file 2

```
Km-Y1             CTGTGTGTAACGGTTATATTTCGTTTTTTATTTTTTTTC--CACTGCCATAGAGAAAGAA -76
Km-ATCC12424      CTGTGTGTAACGGTTATATTTCGTTTTTTATTTTTTTTC--CATTGCCATAGAGAATGAA -78
Km-CBS834         CTGTGTGTAACGGTTATATTTCGTTTTTTATTTTTTTTC--CATTGCCATAGAGAAAGAA -80
Km-DMKU3-1042     CTGTGTGTAACGGTTATATTTCGTTTTTTATTTTTTTTTTCCATTGCCATAGAGAAAGAA -78
Km-CBS6556        CTGTGTGTAACGGTTATATTTCGTTTTTTATTTTTTTTT-CCATTGCCATAGAGAAAGAA -78
                  **************************************   ** ************ ***

Km-Y1             AAAAAAAA---GAGAGT----GATGATCTTCCATTCGAATCCCATAAGTGATACTTTT-- -25
Km-ATCC12424      AAAAAAAA---GAGAGT----GATAATCTTCCATTCGAATCCCATAAGTGACACTTTT-- -27
Km-CBS834         AAAAAAAAAA-GAGAGT----GATGATCATCCATTCGAATCCCATAAGTGACACTTTT-- -27
Km-DMKU3-1042     AAAAAAAAAAAGAGAGTTTGTGAAGATCTTCCATTCGAATCCCATAAGTGACACATTTAA -18
Km-CBS6556        AAAAAAAAAAAGAGAGTTTGTGAAGATCTTCCATTCGAATCCCATAAGTGACACATTTAA -18
                  ********   ******    **  *** ********************** ** ***
                                            +1
Km-Y1             TTT--GTTTTATCAATTTAGTTCGAGATG
Km-ATCC12424      TTTTTGTTTTATCAATTTAGTTCGAGATG
Km-CBS834         TTTTTGTTTTATCAATTTAGTTCGAGATG
Km-DMKU3-1042     TTTTTTTTTTGTTAGAT---------ATG
Km-CBS6556        TTTTTTTTTTGTTAGAT---------ATG
                  ***   **** * *  *
```

Additional file 2 (continued)
